# “The stroke has already happened. You can't undo what has happened” emotions, coping, and adjustment in the experiences of southeast Asian people living with stroke - A qualitative study^☆^

**DOI:** 10.1016/j.pmedr.2025.103201

**Published:** 2025-08-13

**Authors:** Pablo Cruz Gonzalez, Tingyuan Koh, Matthew Hok Shan Ng, Myra Jasmine Ibrahim, Mun Yu Chan, Eloise Lie, Karen Sui Geok Chua, Phyllis Liang

**Affiliations:** a Rehabilitation Research Institute of Singapore, Nanyang Technological University, Singapore; b Lee Kong Chian School of Medicine, Nanyang Technological University, Singapore; c Institute of Rehabilitation Excellence, Tan Tock Seng Hospital, Singapore; d Yong Loo Lin School of Medicine, National University of Singapore; e Department of Epidemiology and Preventive Medicine, Tan Tock Seng Hospital, Singapore

**Keywords:** Stroke, Post-stroke adjustment, Coping, Mental health, Qualitative analyses, Asian, Singapore

## Abstract

**Objective:**

People living with stroke (PLWS) must cope effectively to facilitate the ideal conditions for adjustment and rehabilitation. Cultural values can greatly shape how an individual may cope and adjust to these changes. Considering the absence of published literature documenting how PLWS cope in the local Singaporean context, it becomes crucial to explore the emotional dimensions and cultural factors that shape their experiences. This study aimed to delve into the emotional aspects of PLWS, elucidating the lived experiences and understanding their coping approaches for post-stroke adjustment.

**Methods:**

This study used a qualitative phenomenological approach with semi-structured interviews in Singapore (February 2020–March 2022) among twelve first-stroke participants discussing life changes, emotions, and coping strategies post-stroke. Thematic analysis was conducted.

**Results:**

Four main themes emerged: 1) Internal emotional experiences amidst adversity and uncertainty; 2) Relational experiences of dependency and external support; 3) Age-related differences in experiences of peer support; 4) The importance of self.

**Conclusions:**

PLWS in Singapore face unique emotional challenges influenced by local cultural values, emphasizing self-responsibility and self-management. Younger individuals struggle with unmet social and employment needs, while older individuals experience fewer such issues. Enhancing mental health support, promoting self-management, and increasing employer awareness could improve emotional adjustment and coping in post-stroke recovery. Addressing both psychosocial and cultural factors through targeted interventions and policy initiatives could help prevent post-stroke-related medical conditions in Southeast Asia. A comprehensive approach integrating holistic healthcare, supportive workplace policies, and community-based resources may facilitate better post-stroke adjustment and, in turn, improve long-term health outcomes.

## Highlights

• Individualized support aids emotional well-being in persons living with stroke.
• Fostering inner resilience empowers persons living with stroke to face adversity.
• Age-tailored stroke services address distinct needs of younger and older adults.
• Discharge guidance on self-management supports recovery after stroke.
• These strategies may prevent complications and secondary health conditions.

## 1 Introduction

The unanticipated nature of stroke is responsible for significant changes in people living with stroke (PLWS) (Gray et al., 2014). Many experience physical and cognitive impairments, which present significant barriers to activities of daily living and functioning (Della Vecchia et al., 2019; Sarre et al., 2014). These impairments might become long-standing sequelae, creating a permanent chasm between the individual's past and present selves, known as biographical disruption which often coexists with emotional distress, posing a challenging management endeavour (Pallesen, 2014; Satink et al., 2013). PLWS commonly experience emotions such as fear, frustration, anger, and grief in response to the resulting disabling conditions (Theadom et al., 2019). Over time, emotional distress can lead to mood disorders such as depression, anxiety, and apathy, affecting at least 30 % of PLWS (Hackett and Pickles, 2014).

In Singapore, poor mental health post stroke is concerning, with post-stroke depression (PSD) affecting 19.24 % of inpatients with ischemic stroke, and 24.59 % of those with haemorrhagic or strategic basal ganglia/thalamic strokes. Among 547 patients followed up within 12 months, PSD occurred in 6.42 % of ischemic, 3.52 % of haemorrhagic, and 5.23 % of strategic strokes (Chan et al., 2024). The same study reported high dropout rates from a local PSD screening initiative involving community-dwelling stroke survivors, suggesting ongoing barriers to mental healthcare access despite the prevalence of PSD (Chan et al., 2024).

The stroke care pathway in Singapore outlines immediate management, secondary prevention, and rehabilitation (Venketasubramanian et al., 2011). While rehabilitation referrals are standard practice, addressing mental health needs often relies on the discretion of the primary care team, including doctors, nurses, and therapists, on a case-by-case basis. Although these healthcare practitioners have the potential to support the mental health of patients with stroke and help them adjust to their new condition, proactive engagement in mental health management is not consistently practiced across the board. This issue is not unique to Singapore; similar gaps have been reported in Western healthcare systems, where greater emphasis is often placed on physical rehabilitation, and calls have emerged to better address the so-called “invisible” consequences of stroke (Liang et al., 2025). Given that more than half of PLWS do not regain the pre-stroke level of functioning (Hankey et al., 2002), both physical and psychological adjustments are essential to the rehabilitation process.

Adjustment commonly refers to a process whereby individuals adapt to and learn from the broad changes brought about by significantly life-altering circumstances (Brennan, 2001). Studies examining experiences of stroke patients (Satink et al., 2013; Liang et al., 2021) have highlighted themes such as the loss of identity and difficulties in developing a “new self” post-stroke. A systematic review (Sarre et al., 2014) identified personal characteristics, adjustment strategies, relationships and structural issues as central factors affecting adjustment outcomes in stroke. However, the current research examining adjustment post-stroke has highlighted the subjective experiences of PLWS from Western regions (Della Vecchia et al., 2019; Liang et al., 2017; Smith et al., 2021).

Singapore's multi-ethnic population embodies a rich diversity of cultural beliefs and practices surrounding illness, disability, and recovery (Pang et al., 2018), which can shape emotional experiences and expressions post-stroke. A local qualitative study, for example, highlighted family-mediated decision-making as a distinctive health-seeking pattern in Singapore's socio-cultural context (Tyagi et al., 2021). More broadly, cultural values are known to influence coping strategies and health-related behaviours (Kuo, 2013). Among East Asian immigrants, conflicting beliefs with Western medical norms have been linked to poor adherence in type 2 diabetes management (Park et al., 2016). Despite such evidence from other health domains, there remains a lack of research investigating the adjustment process of the “new self” post-stroke in the Singapore context. This gap aligns with the inconsistency in how mental health aspects of stroke are practised within local healthcare. The present study aims to fill this gap by investigating the emotional aspects of being an individual with stroke, elucidating their lived experiences, and exploring the coping approaches associated with the aftermath of a stroke in Singapore.

## 2 Materials and methods

### 2.1 Study design and ethics

A qualitative phenomenological approach (Giorgi, 2009) was employed to understand the lived experiences PLWS, focusing on their coping and adjustment processes. Phenomenology explores how individuals describe and interpret their experiences within their unique context. It assumes that life after stroke can be understood from an existential perspective, aiming to uncover the core essence of these experiences (Creswell and Poth, 2017).

Reporting broadly aligns with the COnsolidated criteria for REporting Qualitative research (COREQ) guidelines (Tong et al., 2007). This study was approved by the NTU Institutional Review Board, reference IRB-2019-10-023 and was conducted in accordance with the ethical principles of medical research outlined in the Declaration of Helsinki. All participants gave written informed consent prior to interviews. The study information emphasized that confidentiality was guaranteed, and the transcribed interviews were de-identified and pseudonymized.

### 2.2 Sampling strategy and participants

This qualitative study was nested within a larger investigation of post-stroke quality of life (Ibrahim et al., 2022). Preliminary analysis revealed significant narratives on emotional coping and post-stroke adjustment, highlighting the need for further exploration. Participants were purposively sampled based on these expressed narratives during the interviews and recruited through two clinical sites with a recruitment poster. To gain a broader range of experiences, participants were recruited along the entire recovery continuum. All participants were individuals >21, experiencing a first-ever stroke (regardless of time since onset), with adequate communication and cognition (full score on the adapted Abbreviated Mental Test) and no psychiatric history. Researchers had no prior relationship with participants.

### 2.3 Data collection

A semi-structured interview guide was developed from the literature and research team input, covering concepts of post-stroke quality of life and lived experience (Table 1). The interviews were conducted individually, either online via audio or video calls (*n* = 10), or in person (*n* = 2) at a conducive location. Data was collected by experienced female qualitative researchers (PL, MI, MYC, EL) with backgrounds in psychology and occupational therapy. The interviewers were predominantly of Chinese ethnicity, with one of Malay ethnicity. The Chinese interviewers were proficient in both English and Mandarin, while the Malay interviewer was fluent in English and Malay. Two researchers had substantial clinical experience working with the stroke population, and one also had prior research experience in this area. Interviews were audiotaped, with each lasting approximately one hour. Interviews conducted in English (*n* = 10) and those conducted in Mandarin (*n* = 2) were transcribed, and then translated to English, and then back-translated to Mandarin to check for accuracy.

**Table 1:** Semi-structure interview guide exploring concepts of post-stroke quality of life and lived experience among adults living with stroke in Singapore (February 2020–March 2022).

Questions
1. Tell me about your life after you have sustained a stroke.
2. Tell me about the changes you have noticed in your everyday life.
3. Tell me about how you are coping or feeling.
4. How do you spend your time now? Tell me about the changes since you have sustained a stroke till now.
5. Tell me about your relationship with your family members.
6. Tell me about the things you want to achieve and your goals.
7. Tell me about the supports or help you have received and how you feel about it.
8. What do you think may help you now? Tell me your expectations of being supported.
9. Tell me about the positive experiences you have had since you sustained a stroke.

After each interview, a summary of the key points was sent to participants for verification, enhancing the qualitative rigour. Six participants confirmed the summaries accurately reflected their interviews. Five provided minor edits and/or additional insights, which were incorporated into the transcripts prior to analysis. One participant did not respond. To further ensure trustworthiness and credibility, researchers bracketed their preconceived beliefs, biases, and notions (Giorgi, 2009) and engaged in reflexive reflection after each interview.

### 2.4 Statistical analysis

Voice recordings of interviews were transcribed verbatim, and transcripts were inductively analysed by Giorgi's approach of analyses (Giorgi, 2009) which prioritises individual-level analysis before synthesising meaning across participants. This approach ensures that the integrity of each participant's lived experience is preserved while uncovering shared themes (Giorgi, 2009). The process was iterative and began with multiple reviews of transcripts for familiarisation. Generating initial codes involved systematically categorising data. Next, codes were collated into potential themes and reviewed. This finally led to the synthesis of the data as the themes were refined and defined. Audit trails of analytical decisions were used to ensure dependability. Analysis was conducted to the point of data sufficiency (Saunders et al., 2018) by female researchers with backgrounds in psychology, occupational therapy and medicine. Data was primarily analysed by TY, MI and EL and then explored with PC and MN to achieve consensus on the interpretation of the data. PL and KC provided guidance throughout process. The researchers determined that analysis of data from 12 participants yielded sufficient thematic insights to support code clarity and theme development. NVivo (QSR International, version 13, 2020) software was used to facilitate the analysis.

## 3 Results

Twelve participants (9 females; mean age = 56.17, SD = 15.41; range 26–79 years). Four out of twelve participants were younger individuals, aged <55 years. Time post-stroke ranged from 4 months to 17 years (mean = 5.53 years, SD = 5.00), with 11 classified as living with chronic stroke (>6 months post-stroke; Table 2).

**Table 2:** Basic demographics and functional characteristics of adults living with stroke in Singapore (February 2020–March 2022).

Case	Gender	Age	Time since stroke	Ethnicity	Marital status	Main caregiver	Employment status (current)	Financial situation (Household income monthly)	Basic activities of daily living status (current)	Instrumental activities of daily living Status (current)	Indoor mobility	Outdoor mobility
1	Female	56	17 years	Chinese	Married	Self	Admin executive	$4000–$4999	Require help	Independent	Independent	Independent
2	Female	58	6 years	Chinese	Single	Helper	Unemployed	$2000 -$2499	Independent	Require help (maid to do the lifting)	Independent (wheelchair and broad based quad stick)	Require help (motorised wheelchair)
3	Female	66	4 months	Chinese	Married	Helper	Semi-retired	$10,000- 12,499	Require help	Require help	Supervision	Supervision
4	Female	52	3 years	Chinese	Married	Mostly helper, husband	Unemployed	$2000 - $2499	Require help	Require help (broad based quad stick)	Require help (broad based quad stick)	Supervision (broad based quad stick)
5	Female	60	2 years	Chinese	Single	Helper	On medical leave	$5000–$5999	Independent	Independent	Independent	Independent
6	Female	79	7 years	Chinese	Widowed	Self	Self employed	$2000 or less (earning about $200–300, the rest is savings & investments)	Independent	Supervision	Independent	Supervision
7	Male	57	10 years	Chinese	Married	Wife	Self-employed	$6000 - $7999	Independent	Independent	Independent	Independent
8	Female	26	1 year	Chinese	Married	Self, husband, mother-in-law	Unemployed.	$2500	Independent	Requires help for child rearing and housekeeping	Independent	Supervision
9	Female	40	11 years	Chinese	Single	Mum	Unemployed	$2000 - or less (no income)	Require help	Require help	Independent	Independent (quad stick)
10	Female	68	2 years	Chinese	Married	Husband	Retired	$2000 - or less (no income)	Independent	Requires help for heavy lifting	Independent	Independent
11	Male	38	2 years	Chinese	Married	Wife	Unemployed	$2000 or less	Independent	Independent	Independent	Independent
12	Male	74	5 years	Chinese	Married	Helper	Unemployed	0$ rely on savings	Require help	Require help	Require help	Require help

$, Singapore Dollars.

Four main themes were identified (Table 3): 1) Internal emotional experiences amidst adversity and uncertainty; 2) Relational experiences of dependency and external support; 3) Differing adequacy of age-appropriate support for younger versus older individuals with stroke; 4) The importance of self.

**Table 3:** Themes, subthemes and illustrative quotes on post-stroke coping and adjustment among adults living with stroke in Singapore (February 2020–March 2022).

Theme	Subthemes	Illustrative Quotes
1. Internal emotional experiences amidst adversity and uncertainty	Fear	“I'm very afraid that I will have a second stroke and end up like that.”
	Frustration	“I get frustrated because I don't think I can do the things by myself.” “I really feel very useless, I can no longer do even basic things like eating or going to the toilet.”
	Sadness	“Little bit cry and then little bit cry, then want to kill yourself a little bit.” “When I first came home, I cannot walk, I cannot do anything, I wanted to jump down from my house.” … “Because I thought after the cranioplasty everything should get better right? But everything is the same.”
	Worry	“When I take the public transport, I have this worry in my heart, don't know whether can make it or not.”
	Gratitude	“I've managed to achieve more personal growth because of the stroke and I'm glad for it, I have different sets of challenges, I might not achieve the same that I have right now.”
	Hope	“After you gradually undergo more treatment, you will feel that there are many things that you can slowly become better at, you will be able to do.” “Don't lose hope don't be discouraged. Just continue to exercise” … “I just let it flow naturally and just hope that I recover as soon as possible”
2. Relational experiences of dependency and external support	Emotional and moral support	“My husband left his job because when I was discharged from my hospital I couldn't accept it, so I was in depression so I always like try to attempt suicide so he stayed and looked after me during that first year he would encourage me you know, when I'm very sad you know, when I start to grumble about this thing I cannot do that, I get very upset then he will know how to comfort me.” “It touched me when I cry, my husband also cries with me. My husband was telling me, ‘You cannot go and just throw me behind. We must walk together.”
	Medical support	“This psychology doctor was very good, he encouraged me to move on. He was telling me that, ‘A lot of things can be fixed, whether your mood, if you have financial problems, we can help you to fix it. We can get a social welfare to fix it. If you have walking problem, we can get a rehab doctor to help you to fix it. You must stay happy then you can do a lot of things.’ Initially, I was on very high dosage of depression medication. After that, I don't need them anymore. I manage to walk out of my depression.”
	Rehabilitative support	“The rehab department helped me a lot. They trained me how to sit, stand, squat.” “My therapist was very good, he taught me how to walk with walking stick, that moment I was very happy, really.” “Very good. I can discuss with [my physiotherapist and occupational therapist] whatever my sorrows and my happy and not happy. They will encourage me to move on.” “The therapist that is very helpful really changed my mood a lot. I met two very helpful therapist to help me do a lot of things. One of the therapist even helped me to go online to apply for my house to be installed with all the handicapped bars.”
	Feeling like a burden	“They are actually all very busy. So, I really feel bad.” “I don't like to rely on people. I like to do things myself you see then I don't like to be a parasite to other. I feel bad even, even they said, ‘It's alright, it's alright’ I still feel bad.”
3. Age-related differences in experiences of peer support	Adequate support	“And having a group of friends that share the same thing, those stroke friends that I have, it also helps because we can confide in each other and help each other” “Because we are stroke, they are stroke, we know what we are going through.”
	Inadequate support	“There was this gap there that didn't exist something like a support group in that sense for younger stroke survivors because our challenges are so much different than an older stroke survivor.” “All the stroke survivors there are elderly then when you see that you are the youngest survivor there you will keep thinking Why did I get stroke?
4. The importance of self	Accepting reality	“The stroke has already happened so the only thing you can do is work towards what you want, the goals that you wish to achieve, you cannot undo what has happened regardless of how much you complain, the next best thing you can do is accept.” “Bad things happen sometimes and we just have to move past it and carry on because that is just life.”
	Being internally driven	“If I want to recover, it's all about me only. Nobody else. Nobody else can do it. So, in my mind, it has always been, I have to do it. I have to recover. I have to get back to normal.” “The doctor will give you advice, the physio will give you advice, but you have to do it yourself. You don't help yourself nobody can help you.”
	Persevering amidst hardship	“I'm a survivor type. I have the fighting spirit in me.” “It's never-ending stroke, if you stop means you may get suddenly you will get even worse, if you stop moving or exercising your muscle will get harden or stuff. So, you keep on you need to keep moving, keep moving. When you stay alive then you can keep moving.”
	Taking the first step into community	“You have to be in the community, you have to go in. You don't wait outside, you know.”

### 3.1 Internal emotional experiences amidst adversity and uncertainty

In response to post-stroke changes and challenges, participants described a range of internal emotional experiences, including frustration, sadness, hopelessness, and, for some, eventual hope and personal growth. Most participants stated that they felt useless and incapable. These thoughts often led to negative self-evaluation, triggering feelings and emotions such as frustration and sadness. Impaired motor function hindered individuals from completing simple tasks, fostering a sense of inadequacy and frequent frustration. In addition, the loss of independence led participants to think they could not control their own lives. This barrier to self-empowerment resulted in frustration (Table 3, Theme 1, Frustration subtheme).

All participants experienced sadness shortly after their stroke, realizing recovery would be prolonged and unlikely to fully restore their abilities. This sense of permanent loss often caused despondency (Table 3, Theme 1, Sadness subtheme). However, the duration of sadness varied among PLWS. Some participants only experienced sadness for the initial months post-stroke. Others continued to experience sadness even after more than 10 years.

“I was low period for I think seven over months.” (Elizabeth, 52).

“Every time I pass by a group of people dancing, memories come back and made me very sad.” (Sandra, 56).

In addition, a common cause of sadness among younger participants was lagging behind their peers in career progress and social activities, resulting in missing out on what others their age were experiencing.

“Everyone my age is leading very normal lives, working, eating and drinking outside but instead I have to use the time to do rehabilitation. So, I will feel very, very sad, like why does it have to be me.” (Pamela, 26).

Meanwhile, older participants were more likely to experience sadness because of challenges in physical recovery.

“I had improved a bit, but I still have this imbalance.” … “When I think about my condition, I feel very sad.” (Jasmine, 68).

Seven of the twelve participants expressed gratitude for post-stroke challenges, viewing them as opportunities for self-improvement and resilience they might not have otherwise developed (Table 3, Theme 1, Gratitude subtheme). Some PLWS also experienced hope as they noticed physical improvements during rehabilitation, believing in their potential to regain more function (Table 3, Theme 1, Hope subtheme).

### 3.2 Relational experiences of dependency and external support

Alongside their internal emotional struggles, participants highlighted how their recovery journey was shaped by external relational dynamics, particularly their dependence on family and healthcare providers. While these support systems were essential, they also triggered emotional tension.

In the initial six months, participants primarily relied on external support as they had difficulty managing their emotions that resulted from the aftermath of the stroke. Family members provided emotional and moral support (Table 3, Theme 2, Emotional and moral support subtheme).

Most participants did not initially expect their family members to take on caregiving responsibilities. However, for those who had family around, family members often stepped in to help.

“I like to do things myself you see then I don't like to be a parasite to other. I feel bad even they said, ‘It's alright, it's alright,’ I still feel bad. I know where is my limit and I set my limits” (Aly, 79).

“Yeah and that helped a lot trying to be more independent and showing that I was not totally dependent on her give her a peace of mind to carry on her work because I also did a lot at times she was actually travelling quite a lot” (Nathan, 38).

While this support was appreciated by most, two participants expressed a gap in caregivers' ability to be attuned to their emotional needs.

PLWS also relied on healthcare professionals for medical care, while therapists provided them with supportive rehabilitation. In addition to regaining physical skills, participants described how rehabilitation supported their emotional well-being (Table 3, Theme 2, Rehabilitative support subtheme).

However, some PLWS expressed that increased dependence on loved ones had the potential to lead to guilt. They perceived themselves as a burden, taking up the time and resources of others (Table 3, Theme 2, Feeling like a burden subtheme).

### 3.3 Age-related differences in experiences of peer support

Older PLWS found comfort through interactions with other PLWS. The sudden and often immense loss in physical and cognitive function post-stroke left individuals with many complex emotions that were not the easiest for others to understand and relate to. The validation of their struggles by other PLWS was able to bring about solace (Table 3, Theme 3, Adequate support subtheme).

A minority of younger PLWS even stated that being with other PLWS, who were mostly older, made them feel worse about themselves. They felt singled out (Table 3, Theme 3, Inadequate support subtheme).

### 3.4 The importance of self

After the initial six months of recovery during which they relied primarily on external support, PLWS began to cope through self-effort. While they acknowledged the external support, they expressed that the role of self was most important (Table 3, Theme 4, Being internally driven subtheme).

The first step in moving forward was accepting what had happened. They had to come to terms with their situation before they could be at peace and focus on working towards their recovery goals (Table 3, Theme 4, Accepting reality subtheme).

While being internally motivated helped PLWS begin their efforts towards recovery, it was perseverance that sustained them throughout the process (Table 3, Theme 4, Persevering amidst hardship subtheme).

## 4 Discussion

**Fig. 1:**
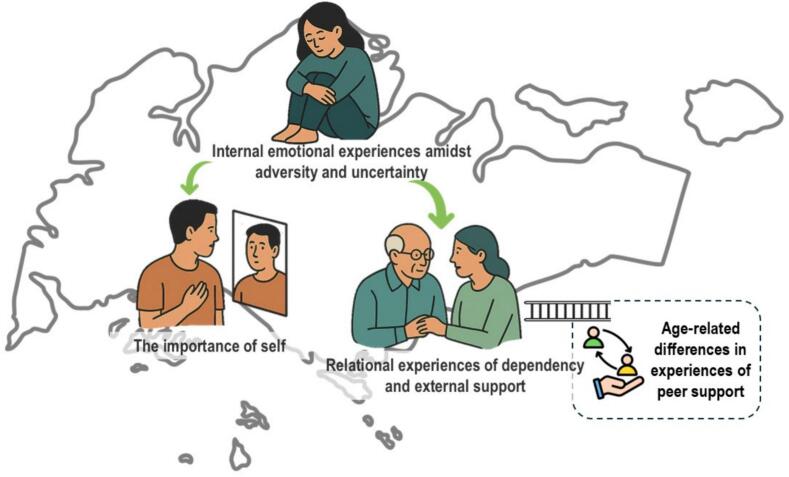
Thematic visualization of themes and their contextual representation of post-stroke coping and adjustment themes among adults living with stroke in Singapore (February 2020–March 2022).

This study aimed to delve deeper into the post-stroke adjustment of PLWS. The discussion section is organized around broader conceptual insights that cut across the identified themes (Fig. 1).

### 4.1 Emotional complexity and cultural influence

This study showed that PLWS experienced many thoughts when faced with changes which shaped their emotional responses. A plausible explanation is that emotions might be considered a reaction to how we assess a situation based on whether it aligns with goals and expectations as well as whether it is easy to control (Moors, 2017). As a result, participants with stroke experience negative emotions because their lives undergo significant disruptions, including altered plans and a loss of predictability and stability. These negative emotions may be particularly pronounced in Singapore, where there is a strong emphasis on personal responsibility for health and a societal expectation to avoid overreliance on the broader community (Olsson et al., 2008). However, this perspective can clash with the cultural expectations in Asian societies, where young family members are traditionally expected to care for the ill and elderly (Speirs et al., 2017). This cultural clash may intensify the negative emotions of PLWS in Singapore, who may feel a sense of inadequacy for not being independent or responsible for their own health. Further, these findings are corroborated by other studies which found that PLWS experienced big negative emotions (Wenzel et al., 2021) related to physical impairment (Kim, 2016). Another cause of negative emotions mentioned by participants is the uncertainty of the future amidst all the changes post-stroke, which is consistent with previous findings that reported fear of the future in PLWS (McCurley et al., 2019). This study also revealed that sadness can last for varying durations among PLWS, possibly due to different mindsets. (Moeller and Carpenter, 2013).

It is worth noting that negative emotions following a major life event such as stroke may be universal and form part of the broader psychological process of coping and adjustment. However, the way these emotions are experienced and managed can be shaped by sociocultural context. In the case of Singapore, the co-existence of cultural values such as self-reliance and filial responsibility may create internal conflicts that heighten emotional strain. This distinction underscores the importance of recognising both universal and culturally specific dimensions of post-stroke adjustment.

While negative emotions are prevalent among PLWS, there are also positive emotions such as hope (Visvanathan et al., 2019). Hope has been shown to play a critical role in stroke recovery because it provides a vision for a better self and provides participants with stroke with the motivation to keep pushing forward in rehabilitation towards one's goals (Soundy et al., 2014). Higher levels of positive emotions are associated with better motor and cognitive function, as well as greater improvement in functional status in participants (Ostir et al., 2008). These emotions provide mental space for new possibilities, fostering motivation for rehabilitation and resilience in overcoming challenges. (Ostir et al., 2008).

### 4.2 Relational experiences of dependency and external support

This study aligns with previous research showing that PLWS rely on emotional and moral support from family, friends and others with the same condition (Visvanathan et al., 2019; Moss et al., 2021) as well as medical support from healthcare professionals (Lo et al., 2023). Such support not only benefited the mental health of PLWS but also helped them persevere in rehabilitation and make progress in physical recovery. However, some PLWS expressed that increased dependence on loved ones led to guilt. This is in line with other studies which showed that PLWS felt uncomfortable (Lynch et al., 2008; Thomas et al., 2018) and guilty (Smith et al., 2021) about the burden they placed on others.

### 4.3 Age-specific challenges and biographical disruptions

This study revealed that the common cause of sadness is different in younger and older individuals with stroke. One of the biggest causes of sadness and anxiety in younger groups is being unemployed. Unique to them is their stage of life, at which most of their peers are steadily building up their careers and growing their financial capabilities. Research has demonstrated that unemployment contributed to a reduced quality of life in younger individuals with stroke (Morris, 2011). Meanwhile, this study found that the psychological well-being of older PLWS is mainly affected by their motor impairment instead of unemployment.

These differences may reflect broader experiences of identity loss, which are often shaped by age-related roles and social expectations. For younger individuals, stroke frequently disrupts emerging adult roles related to employment, financial independence, and peer socialisation. In contrast, older individuals may already be retired or in declining health, and thus the experience of stroke tends to reinforce rather than abruptly alter their social roles. This highlights the need to understand biographical disruption not just in terms of function, but also in relation to life stage and societal expectations.

### 4.4 The evolving role of self

Self-effort is the most reported way by which PLWS cope with their emotions after the initial six months of recovery. This finding aligns with previous studies, indicating that they felt that having the best understanding of their emotions and needs makes the role of self-indispensable in managing their emotional well-being (Pallesen, 2014; Satink et al., 2013; Lo et al., 2023). Thus, goal setting and the process of improving emotional well-being should be centred around the individual with stroke (Kimmel et al., 2020; Nott et al., 2021). In this study, the emphasis on self-effort among PLWS in an Asian society reflects the ongoing shift in Eastern societies towards individualism (Ogihara, 2018). This observation aligns with our earlier discussion on the amplified negative emotions and increased individual responsibility for health and self-reliance, which suggest a growing trend of self-management among Singaporeans with stroke.

It is of note that the first step PLWS had to take was acceptance of what had happened. These individuals had to come to terms with their new and altered reality before they could move on and work towards their goals in recovery. This corroborates previous studies that state that acceptance of disability plays a critical role in the psychosocial regulation and disability adaptation of PLWS (Chai et al., 2016). Such acceptance also reduces the occurrence of psychological conditions such as depression and suicidality (Bartoli et al., 2013).

### 4.5 Implications for healthcare practice

PLWS revealed that rehabilitation positively impacted their emotions and coping abilities through the restoration of mobility, independence training, and encouragement. This, combined with adjustment counselling (White and Johnstone, 2000) is likely to have a synergistic effect. Since positive emotions are beneficial in stroke recovery, there is an opportunity to incorporate considerations for the emotional well-being of PLWS in Singapore. The Singapore Ministry of Health Clinical Practice Guidelines on stroke provide guidance for medical management and physical rehabilitation but there is a glaring absence of guidelines for the coping and adjustment of stroke patients to their condition (Venketasubramanian et al., 2011). Although there are currently some initiatives in public healthcare institutions such as “Consultation-Liaison Interventions for the Mind and Brain” these initiatives have not yet been adopted as the standard of care. Consequently, it can be argued that the adjustment needs of PLWS are often viewed as less important and more of an afterthought as compared to their medical and rehabilitation needs, rather than being integrated as a core component of the rehabilitation process. There is a need to expand the current guidelines to explicitly incorporate strategies and interventions for managing the adjustment process.

Another important area to cover would be emphasizing building inner resilience such as through promoting positive self-image and self-compassion (Gilbert, 2014). Interventions addressing both spheres might be more effective. A key aspect worth discussing is the enhancement of existing age-appropriate services provided by community organizations and social workers. This includes not only expanding the current return-to-work services available in hospitals and the community but also creating age-specific support and social groups. Our findings have revealed that younger PLWS have particularly significant vocational needs compared to older individuals, and addressing these needs promptly during the rehabilitation process may help alleviate anxiety and mitigate feelings of social loss. Additionally, understanding and accommodating employers would play a critical role. Lastly, this study also found that PLWS had difficulty coping through self-effort during the initial transition period post-stroke. Hence when preparing stroke patients for hospital discharge in Singapore, more guidance on self-management of thoughts and feelings (Pearce et al., 2015) could be provided.

### 4.6 Limitations

There is a potential sampling bias, as participants were selected based on the narratives they reported around emotional coping post-stroke, and the study excluded participants with significant communication or cognitive impairments. Additionally, the heterogeneity in the time elapsed since stroke among participants may have influenced their lived experiences introducing variability. Another limitation of this study is the lack of racial diversity among participants, as all were of Chinese ethnicity. Although the inclusion criteria were not restricted by race, the COVID-19 pandemic's social distancing measures made it challenging to recruit participants from diverse ethnic backgrounds. In the context of Singapore's multicultural society, a more racially diverse sample would enhance representativeness and provide a more comprehensive understanding across cultural contexts. For example, cultural differences among racial groups, such as the Malay families' tendency to utilize both formal and informal social support networks compared to Indian and Chinese families, may influence the balance between self-reliance and reliance on social or community resources (Bentelspacher et al., 1994). Despite these limitations, this is the first study to examine post-stroke coping and adjustment in Singapore, providing cultural insights that fill a gap in the predominantly Western literature.

## 5 Conclusions

In the face of post-stroke changes and challenges, individuals experience a range of thoughts that influence their emotions and shape their adjustment process. They cope with these emotional consequences by relying on both external support and personal resilience. Among PLWS, negative emotions may be amplified by local expectations, such as an overemphasis on self-responsibility for health and a reliance on self-effort, reflecting the growing trend of self-management among PLWS in Singapore. Younger individuals often find external support inadequate, primarily due to unmet employment needs, pressures from “sandwich generation” responsibilities, and challenges in social integration.

Expanding healthcare guidelines to include post-stroke adjustment strategies may extend evidence-based mental health practices to more PLWS. A structured approach considering cultural and psychosocial influences could reduce post-stroke health complications. Strengthening healthcare policies, workplace support, and community-based resources may enhance post-stroke adjustment.

## Declaration of funding and conflicting of interest

This work was supported by the Rehabilitation Research Institute of Singapore under PL - RFP/19001 Grant. The funder has played no role in study design, analysis and interpretation of data, or preparation of manuscripts. The authors report there are no competing interests to declare.

## CRediT authorship contribution statement

**Pablo Cruz Gonzalez:** Writing – review & editing, Writing – original draft, Supervision. **Tingyuan Koh:** Writing – original draft, Software, Investigation, Formal analysis. **Matthew Hok Shan Ng:** Writing – original draft. **Myra Jasmine Ibrahim:** Software, Project administration, Formal analysis. **Mun Yu Chan:** Data curation. **Eloise Lie:** Writing – review & editing, Supervision, Project administration, Investigation, Formal analysis, Data curation. **Karen Sui Geok Chua:** Supervision. **Phyllis Liang:** Supervision, Resources, Methodology, Investigation, Funding acquisition, Conceptualization.

## Declaration of generative AI and AI-assisted technologies in the writing process

ChatGPT was also employed for grammar checking, language polishing over the manuscript draft in parts where there were doubts about readability and clarity for the reader, and aiding the authors to summarize. iThenticate was used for plagiarism checking after finalizing the manuscript.

## Declaration of competing interest

The authors declare that they have no known competing financial interests or personal relationships that could have appeared to influence the work reported in this paper.
